# Isolation and Characterization of High-Efficiency Rhizobia From Western Kenya Nodulating With Common Bean

**DOI:** 10.3389/fmicb.2021.697567

**Published:** 2021-09-10

**Authors:** Clabe Simiyu Wekesa, Alexandra C. U. Furch, Ralf Oelmüller

**Affiliations:** Department of Plant Physiology, Matthias Schleiden Institute of Genetics, Bioinformatics and Molecular Botany, Friedrich-Schiller-University Jena, Jena, Germany

**Keywords:** rhizobia, comparative genomics, nitrogen fixation, common bean, *Rhizobium phaseoli*, pangenome, synteny blocks, species delimitation

## Abstract

Common bean is one of the primary protein sources in third-world countries. They form nodules with nitrogen-fixing rhizobia, which have to be adapted to the local soils. Commercial rhizobial strains such as *Rhizobium tropici* CIAT899 are often used in agriculture. However, this strain failed to significantly increase the common bean yield in many places, including Kenya, due to the local soils’ low pH. We isolated two indigenous rhizobial strains from the nodules of common bean from two fields in Western Kenya that have never been exposed to commercial inocula. We then determined their ability to fix nitrogen in common beans, solubilize phosphorus, and produce indole acetic acid. In greenhouse experiments, common bean plants inoculated with two isolates, B3 and S2 in sterile vermiculite, performed better than those inoculated with CIAT899 or plants grown with nitrogen fertilizer alone. In contrast to CIAT899, both isolates grew in the media with pH 4.8. Furthermore, isolate B3 had higher phosphate solubilization ability and produced more indole acetic acid than the other two rhizobia. Genome analyses revealed that B3 and S2 are different strains of *Rhizobium phaseoli*. We recommend fieldwork studies in Kenyan soils to test the efficacy of the two isolates in the natural environment in an effort to produce inoculants specific for these soils.

## Introduction

Common bean (*Phaseolus vulgaris* L.) is an essential source of proteins, carbohydrates, vitamin B complex (riboflavin, thiamine, niacin, and folic acid), and vital minerals in human nutrition. Flavonoids and isoflavonoids, mainly produced as defense compounds against phytopathogens, and antioxidants as protectors against UV radiations act as anti-cancer agents that inhibit the tyrosine kinase, cyclooxygenase, protein kinase C, and lipoxygenase enzyme activities (Romero-Arenas et al., 2013). Common bean is therefore not only a source of essential nutrients but also has medicinal features.

In many parts of the world, the production of common bean is restricted by poor soil fertility, particularly nitrogen limitations (Simon et al., 2014). The farmers cannot compensate for this shortage by applying fertilizers, mainly because of economic reasons (cf. Khonje, 1989; Onim, 1993; Savci, 2012). Soil inoculation with rhizobia is an inexpensive and environmentally friendly alternative. Rhizobia not only supply the plants with nitrogen through nitrogen fixation but also protect them against pathogens by activating their immune system (Mishra and Singh, 2006; Beardon et al., 2014). They also confer tolerance to extreme temperatures, drought, salinity, or non-appropriate soil pH, often restricting common bean yield (Mabrouk et al., 2018). Another feature of the rhizobia is phosphorus provision to the host plants through phosphate solubilization (Hao et al., 2014); however, solubilizing phosphate’s ability differs substantially among rhizobial strains.

Kenya is one of the significant common bean producers in East Africa (Katungi et al., 2009; Kawaka et al., 2014; Duku et al., 2020). However, little is known about their local rhizobial communities. Most commercial rhizobia inoculants used in Kenya originate from the United States or South America (Kawaka et al., 2014), such as the widely used *Rhizobium tropici* CIAT899 strain from Colombia. Although this strain is genetically stable and tolerates high temperatures, it does not improve the yields in most Kenyan soils due to low adaptation to local edaphic conditions (Mwenda et al., 2018). Furthermore, it competes with indigenous rhizobia (Mungai and Karubiu, 2011), which are not well characterized but adapted to the local Kenyan conditions. In some circumstances, newly introduced rhizobia were outcompeted by the local strains (Mathu et al., 2012).

Furthermore, the rhizobial community associated with the legumes differs substantially in different agricultural areas. Therefore, isolation and characterization of well-adapted rhizobia related to common bean in Western Kenyan soils are significant initial steps toward developing an inoculant with the potential to improve crop productivity in this region. As an initial attempt, we (1) isolated rhizobia associated with common bean indigenous to Western Kenyan soils, (2) screened for their symbiotic potential, (3) sequenced their genomes, and (4) identified and characterized the isolates by genome analyses.

## Materials and Methods

### Isolation of Rhizobia

Nodules were sampled from the farmers’ field in two regions of Western Kenya with no history of commercial or agricultural rhizobia inoculation: Bukhayo West (0°27′00.2″ N 34°07′59.9″ E) in Busia County and Sang’alo (0°21′04.9″ N 35°03′00.3″ E) in Bungoma County. Western Kenya is generally warm and wet throughout the year. The temperature ranges from 19.6 to 21.9°C and averages 1,395 mm of rain per year, running from 65 to 210 mm per month. Five healthy nodules per plant were carefully removed from randomly selected 10 plants per sampling site and surface sterilized in 70% ethanol for 30 s and 5% sodium hypochlorite for 5 min, followed by seven sterile distilled water changes. They were then crushed in a drop of saline solution, and the exudate was streaked on yeast extract mannitol agar (YMA) and maintained at 30°C for 4 days. Pure colonies were then stored in yeast extract mannitol broth (YMB) at 4°C for short time storage or in YMB with 25% glycerol at −80°C for long-term storage. Plant infection assay (Somasegaran and Hoben, 2012), in which the isolates were re-infected into the common bean plants growing in the sterile vermiculite supplied Leonard jar assemblages (Trung and Yoshida, 1983) and monitored for nodulation ability, was performed to identify rhizobia among the isolates.

### Potential for Nitrogen Fixation, Phosphorus Solubilization, and Indole Acetic Acid Production

#### Nitrogen Fixation

Rosecoco bean seeds (Kenya Seed Company Ltd., Kitale, Kenya) of uniform size were surface sterilized with 70% ethanol and 2% mercuric chloride for 1 min, followed by thorough rinsing with sterile distilled water. Three seeds were planted in plastic jars supplied with sterile vermiculite (Raiffeisen Gartenbau GmbH & Co. KG, Erfurt, Germany). After germination, they were thinned to one seedling per jar. Seven-day-old seedlings were inoculated with 1 ml of the rhizobial culture (*OD*_600_ = 0.5). The experimental setup consisted of five treatments and six replications: (i) plants irrigated with nitrogen solution (0.8 mM NO_3_^–^), (ii) plants inoculated with standard *R. tropici* CIAT899, (iii) plants inoculated with rhizobia isolate B3, (iv) plants inoculated with rhizobia isolate S2, and (v) negative control, no nitrogen nor rhizobia inoculation. The experiment was arranged in a randomized block design. Besides, plants inoculated with the standard *R. tropici* CIAT899 strain were used as a reference. All seedlings were kept moist by a regular supply of nitrogen-free nutrient solution (Beck et al., 1993) consisting of 1.00 mM CaCl_2_⋅2H_2_O, 0.50 mM KH_2_PO_4_, 0.25 mM MgSO_4_⋅7H_2_O, 0.25 mM K_2_SO_4_, 1.00 μM MnSO_4_⋅H_2_O, 0.30 μM H_3_BO_3_, 0.50 μM ZnSO_4_⋅H_2_O, 0.20 μM CuSO_4_⋅5H_2_O, 0.01 μM NaMoO_2_⋅H_2_O, 0.01 μM CoSO_4_⋅7H_2_O, and 10.00 μM Fe citrate. After 45 days, the plants’ total dry weight, total nitrogen, chlorophyll, and carotenoid contents were determined. Dry weight was determined after drying the plant material at 60°C for 48 h in an oven (Heraeus Deutschland GmbH & Co. KG, Hanau, Germany). The N content per plant was analyzed using the phenol-nitroprusside method (Bilbao et al., 1999). For determining the chlorophyll and carotenoid contents, 100 mg of plant material was crushed in 10 ml of buffered acetone (acetone and sodium phosphate buffer in the ratio 4:1), centrifuged at 13,000*g*. Absorption intensity was measured at 663, 646, and 480 nm with Shimadzu UV 160A spectrophotometer (Shimadzu Corporation, Kyoto, Japan). The absorption values at 750 nm were subtracted from the 663, 646, and 480 nm absorptions. We calculated the chlorophyll and carotenoid contents using the equations by Porra et al. (1989) and Sarker et al. (2014), respectively. One-way analysis of variance followed by Tukey’s HSD in Python 3.8 statsmodels package (Seabold and Perktold, 2010) was performed to check if the observed differences in the mean values of biomass, nitrogen, chlorophyll, and carotenoid contents were statistically significant. The results were visualized by the Matplotlib package (Barrett et al., 2005) in Python 3.8.

#### Phosphorus Solubilization

The isolates’ ability to solubilize phosphorus was assayed on agar plates containing Pikovskaya’s media (Pikovskaya, 1948) supplemented with 5 g/l of tricalcium phosphate (TCP) (Sigma-Aldrich, Munich, Germany) and 1.5% agar. After 10 days of incubation at 30°C, the formation of a hallo zone was evidence of phosphorus solubilization ability. We determined the quantity of solubilized phosphorus in the National Botanical Institute Research Phosphate (NBRIP) broth. About 1 ml of bacterial culture (*OD*_600_ = 0.5) from the isolates were washed in sterile 0.85% sodium chloride and then transferred to 100 ml NBRIP media (Nautiyal, 1999) supplemented with 2.5 g/l of hydroxyapatite (Sigma-Aldrich, Munich, Germany), incubated at 30°C and 150 rpm in 250-ml flasks. After 7 days, 10 ml of the broth was withdrawn to determine the amount of solubilized phosphorus by the molybdenum blue assay (Murphy and Riley, 1962) and the supernatant’s pH.

#### Indole Acetic Acid Production

Isolates were grown in 50 ml minimal media at 30°C, which contained CaCl_2_⋅2H_2_O (1,000 μM/l), MgSO_4_⋅7H_2_O (500 μM/l), KCl (50 μM/l), FeEDTA (25 μM/l), KH_2_PO_4_ (3,000 μM/l), H_3_BO_3_ (10 μM/l), MnSO_4_⋅H_2_O (1 μM/l), ZnSO_4_⋅7H_2_O (0.5 μM/l), CuSO_4_⋅5H_2_O (0.1 μM/l), Na_2_MoO_4_ (0.025 μM/l), CoCl_2_⋅6H_2_O (0.005 μM/l), sodium glutamate (1.8 g/l), mannitol (10 g/l), and L-tryptophan (2 g/l). Sodium glutamate and L-tryptophan were supplied as filter sterilized solutions. Indole acetic acid (IAA) was determined by a colorimetric method (Gilbert et al., 2018). Briefly, after 4 days, 1 ml of the broth was centrifuged at 10,000 rpm for 10 min and added to 2 ml of Salkowski reagent (10 mM FeCl_3_ and 35% perchloric acid). The resultant mixture was incubated at room temperature in the dark for 30 min before its intensity was measured at 530 nm. The total amount of IAA was estimated from the standard indole acetic acid curve.

### Phenotypic and Biochemical Characterization of the Isolates

The bromothymol blue (BTB) reaction (Dupree and Wilcox, 1977) was performed to determine the influence of the isolates on the pH of the media. In YMA plates, 0.0025% BTB (Sigma-Aldrich, Munich, Germany) was included. Gram staining was performed following the protocol of Coico (2006), and the stained smear was viewed with Axio Imager.M2 (Zeiss Microscopy GmbH, Germany). Isolates’ ability to absorb Congo red (Carl Roth, Karlsruhe, Germany) was determined following the procedure of Kneen and LaRue (1983). Catalase test was performed by transferring 1 ml of overnight culture broth in 3% hydrogen peroxide. The formation of effervescence was positive for catalase activity. We tested the ability of the isolates to grow on different carbohydrates provided as the sole carbon source. This test was performed in standard YMA media. Mannitol was replaced by L-arabinose, D-glucose, lactose, D-fructose, D-maltose, sucrose, and mannitol (Carl Roth, Karlsruhe, Germany); sodium glutamate (Tokyo Chemical Industries Co. Ltd, Tokyo, Japan); or D-galactose (Sigma-Aldrich, Munich, Germany). Each isolate was analyzed in duplicates, and the growth at 28°C was recorded every 24 h for 5 days. Isolates’ ability to grow in different pH environments was tested in YMB. About 0.4 ml culture broth of cell density at *OD*_600_ = 0.5 was inoculated in 50 ml YMB at pH 4.8, 7.0, and 9.0 and incubated in an orbital shaker (150 rpm) at 28°C. After 12 h, absorbance at 600 nm was recorded and used to calculate the percentage inhibition of the three isolates at pH 4.8 and 9.0 using pH 7.0 as the reference with the equation in Nadri et al. (2014).

### Whole-Genome Sequencing, Assembly, and Annotation

Genomic DNA was isolated from 3-day-old rhizobia strains following a slightly modified protocol by Wilson (2001) and used to prepare 3-kb DNA libraries. Cells were collected from 5 ml broth by centrifugation at 5,000 rpm for 10 min. The cell pellet was re-suspended in 568 μl of TE buffer, then 2 μl of 2-mercaptoethanol and 30 μl of 10% SDS were added and incubated for 15 min at 37°C. At 65°C, 100 μl of 3M NaCl solution followed by 80 μl of 10% CTAB was added and incubated for 10 min. 624 μl of chloroform–isoamyl alcohol (24:1) was added and centrifuged at 10,000 rpm for 5 min. The supernatant was transferred to a new collecting tube, and an equal amount of phenol–chloroform–isoamyl alcohol (24:24:1) was added and centrifuged for 5 min at 10,000 rpm. The supernatant was transferred to a 1.5-ml tube, and equal volume of isopropanol was added, incubated for 2 min at room temperature, and centrifuged for 5 min at 10,000 rpm. The pellet was then washed twice with 70% ethanol and centrifuged at 7,500 rpm for 5 min. All traces of ethanol were removed, and the pellet was air-dried at room temperature for 10 min then dissolved in 50 μl nuclease-free water.

The DNA was sequenced using Illumina’s MiSeq at Novogene (London, United Kingdom) to obtain 250 base pair paired-end reads. Genome assembly was performed with Unicyler v0.4.8 (Wick et al., 2017), gaps in the contigs filled by abyss-sealer v2.2.8 (Simpson et al., 2009), and contig scaffolding was implemented in RagTag v1.0.1 (Alonge et al., 2019). Gene annotation was done using and RASTtk pipeline on the RAST server v2.0 (Brettin et al., 2015), Prokka v1.13.4 (Seemann, 2014), and PGAP (Tatusova et al., 2016).

### Comparative Genomics

#### Pangenome Analysis

Genome sequences of *Rhizobium phaseoli* (GCA_001664385.1), *Rhizobium etli* (GCA_002119845.1), *R. tropici* (GCA_00033088 5.1), *R. leguminosarum* bv. trifolii (GCA_004306555.1), *Sinorhizobium meliloti* (GCA_000006965.1), *R. grahamii* (GCA_003351175.1), *R. pusense* (GCA_013285525.1), *R. esperanzea* (GCA_001664265.1), *R. laguerreae* (GCA_013004195.1), *R. leguminosarum* bv. viciae (GCA_003351345.1), *R. sullae* (GCA_002812325.1), *R. favelukesii* (GCA_000577275.2), and *Agrobacterium tumefaciens* (GCA_003667905.1) were retrieved from the NCBI database. Together with strains B3 and S2 (cf. below), they were used to cluster the genes into their respective cluster of orthologous groups (COGs) with COGsoft (Kristensen et al., 2010) and OrthoMCL (Li et al., 2003). We used a Perl script in GET_HOMOLOGUES v3.3.2 (Contreras-Moreira and Vinuesa, 2013) to generate the pangenome matrix representing the intersection between COGsoft and OrthoMCL clusters. The pangenome matrix was used to generate the maximum likelihood pangenome phylogenomic tree with IQ-TREE v1.6.12 after determining the best fit model by ModelFinder (Kalyaanamoorthy et al., 2017) in GET_PHYLOMARKERS (Vinuesa et al., 2018) and visualized by FigTree v1.4.4 (Rambaut, 2012). We generated a Venn diagram with jvenn (Bardou et al., 2014) to visualize orthologous gene overlaps in different rhizobial strains.

To perform the species identity of isolates B3 and S2, we determined the average nucleotide identity (ANI) of the isolates with FastANI v1.3 (Jain et al., 2018) and average amino acid identity (AAI) with CompareM v0.1.2 (Parkers, 2014). We also determined the digital DNA–DNA hybridization (dDDH) and genetic distances with GGDC v2.1 (Meier-Kolthoff et al., 2014) referenced to *R. phaseoli*, *R. etli*, and *R. leguminosarum*.

#### Synteny Analysis

A synteny analysis was performed to determine the gene order and rearrangements in the genes of isolates B3 and S2 compared to the genome of *R. phaseoli*. *R. phaseoli* were chosen because an orthologous analysis revealed that the isolates B3 and S2 belong to the same species. We performed the analysis with Sibelia v3.0.7 (Minkin et al., 2013) and visualized the results as chords with Circos v0.69-9 (Krzywinski et al., 2009).

## Results

### Potential for Nitrogen Fixation, Phosphorus Solubilization, and Indole Acetic Acid Production

#### Nitrogen Fixation

**FIGURE 1:**
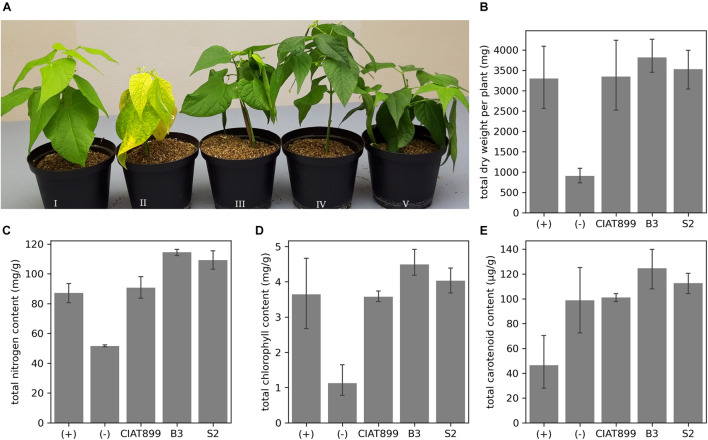
**(A)** The effect of rhizobia inoculation on common bean grown under nitrogen-limited conditions in the greenhouse. Plants supplied with (I) nitrogen fertilizer; (II) no nitrogen fertilizer and no rhizobia; no nitrogen fertilizer but inoculation with CIAT899 (III), B3 (IV), or S2 (V). The **(B)** total dry weight, **(C)** the total quantity of tissue nitrogen, **(D)** chlorophyll, and **(E)** carotenoid content.

We isolated 45 putative rhizobia strains from common bean’s root nodules from two areas in Western Kenya with no commercial rhizobia application history. Reinoculating the bacteria in the common bean identified 10 isolates that generated nodules, which confirmed them as rhizobia. However, eight isolates did not relieve the common bean from acute nitrogen starvation, while two isolates (B3 and S2) did. Inoculation with these two rhizobia allowed the common bean to grow without nitrogen supply, improved plant health (Figure 1A) and yield in terms of total dry weight (Figure 1B) and tissue nitrogen content (Figure 1C). The dry weights of B3- and S2-inoculated plants were significantly higher (*p* < 0.05) than those of the uninoculated plants. They were also more than plants supplied with nitrogenous fertilizer or inoculated with the commercial *R. tropici* CIAT899, though not significant at *p* < 0.05. Moreover, inoculation with B3 and S2 resulted in significantly more tissue nitrogen than in uninoculated, nitrogen-fertilized plants, or those inoculated with CIAT899. The chlorophyll (Figure 1D) and carotenoid (Figure 1E) content was also significantly higher at *p* = 0.05 in plants inoculated with the new strains than uninoculated controls; however, the data was not significantly different compared to the nitrogen-fertilized and CIAT899-inoculated control plants.

#### Phosphate Solubilization and Indole Acetic Acid Production

**FIGURE 2:**
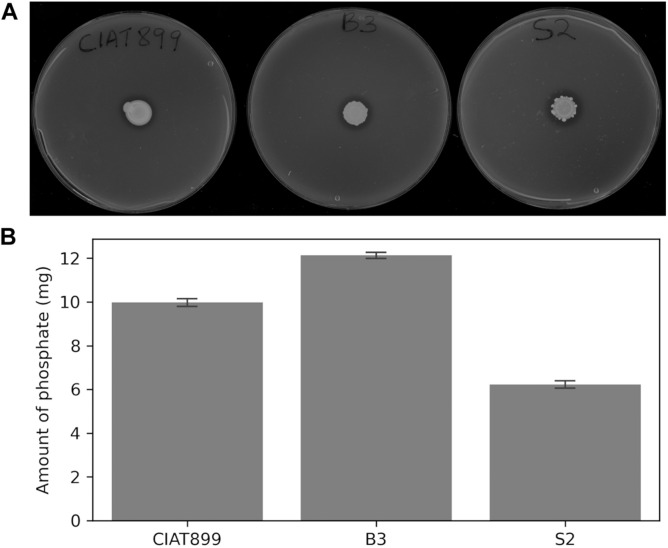
Phosphate solubilization on Pikovskaya’s agar plates **(A)** and amount of solubilized phosphorus in NBRIP broth **(B)** of the rhizobia isolates. Bars represent standard errors based on three independent replications.

The three rhizobial strains, B3, S2, and CIAT899, solubilized insoluble tricalcium phosphate on Pikovskaya’s agar plates as indicated by clear hallo zones around the colonies (Figure 2A) and hydroxyapatite in liquid NBRIP media (Figure 2B). The solubilized phosphate in the medium inoculated with B3 (11.5 mg) was significantly higher than the amounts solubilized by the isolate S2 (7.2 mg) and CIAT899 (10.9 mg) at 95% confidence. Notably, B3 had higher phosphate solubilization efficacy than the commercial CIAT899 strain, and S2 was the least efficacious among the three strains. The supernatants’ pH were reduced from 7.0 to 5.3 for B3 and 5.4 for S2 and CIAT899. Although CIAT899 is known to produce alkaline compounds in full media with pH 7.0, the drop in the pH in media with insoluble phosphate demonstrates that it can also generate acidic compounds if phosphate solubilization is required.

**FIGURE 3:**
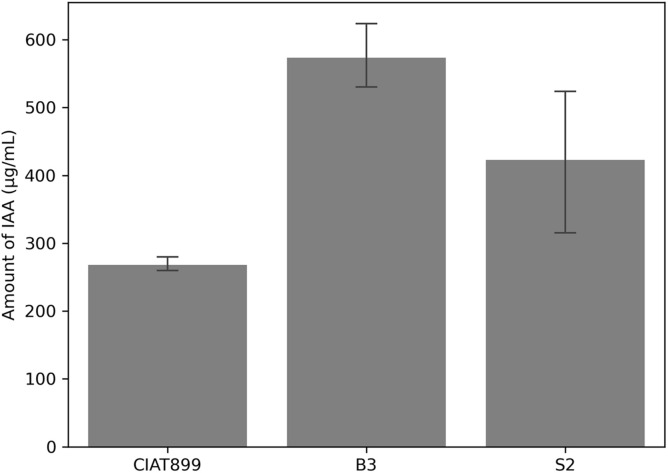
Amount of indole acetic acid (IAA) produced by the isolates CIAT899, B3, and S2 in the liquid media and measured by calorimetric method.

Likewise, the three strains produced IAA in tryptophan-supplemented media. Again, isolate B3 was the most effective IAA producer (574 μg/ml), followed by isolate S2 (423 μg/ml) and CIAT899 (268 μg/ml) (Figure 3). At a 5% error margin, isolates B3 and S2 produced significantly more IAA than CIAT899.

### Phenotypic and Biochemical Characteristics of the Newly Isolated Strains

The isolates B3 and S2 formed colonies with an entire margin, elevated convex surface, and slightly translucent. This was the opposite for CIAT899, whose colonies appeared opaque white and flat on the surface. The B3 colonies appeared more mucoid than S2 and even more than CIAT899, probably due to its overproduction of exopolysaccharides. B3 and S2 changed the green BTB dye to yellow on a complete medium, showing that they are acid producers instead of CIAT899, which changed the color to light blue, indicating that it is an alkaline-producing bacterium (Supplementary Figure 1). Again, different from CAIT899, B3 and S2 only slightly absorbed Congo red (Supplementary Figure 2). The absorption of this dye is often used as a criterion for identifying rhizobia during isolation procedures (cf. section “Discussion*”*).

**FIGURE 4:**
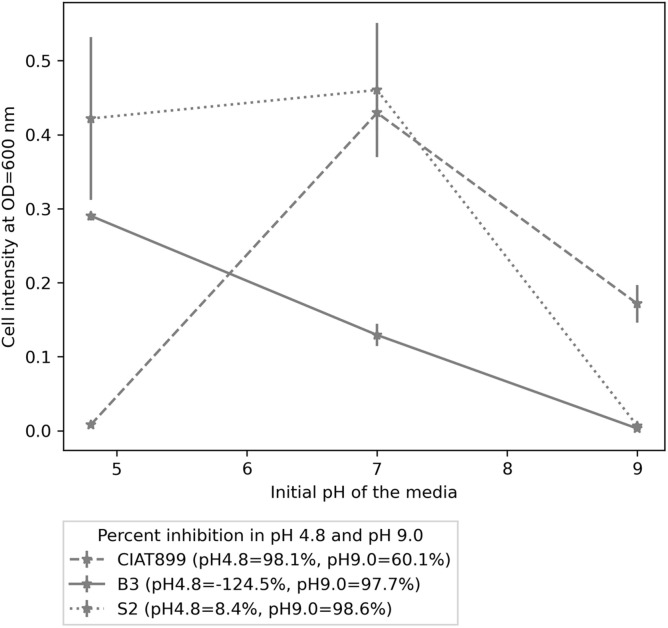
The growth pattern of isolates B3, S2, and CIAT899 in yeast extract mannitol broth (YMB), in which the pH was maintained 4.8, 7.0, and 9.0. The figure legend contains percent inhibition values of the isolates at pH 4.8 and 9.0 when samples at pH 7.0 were used as the reference.

Furthermore, the isolates were stained as gram-negative rods (Supplementary Figure 3), and all three were positive for the catalase test. When applied as sole carbon sources, all isolates utilized mannitol, glucose, glutamate, arabinose, galactose, maltose, fructose, sucrose, and lactose. Isolate S2 and CIAT899 had a maximal growth at pH 7.0, while B3’s maximum growth was observed at pH 4.8. At pH 4.8, CIAT899 was 98.1% inhibited, S2 was only 8.4% inhibited, while B3 was not inhibited. However, all three isolates were inhibited at pH 9.0; CIAT899 performed better in these conditions (60.1% inhibited), while both B3 and S2 were highly inhibited at this pH, 97.7 and 98.6%, respectively (Figure 4).

### Genome Assembly and Annotation

Genomic sequences were obtained from B3 and S2, as described in *Materials and Methods*. Final assembly and contig scaffolding resulted in a single chromosome of about 4.3 Mb and four plasmids of approximately 349 kb (plasmid 1), 376 kb (plasmid 2), 389 kb (plasmid 3), and 1.1 Mb (plasmid 4) for both isolates. The G + C content was 61.5 and 61.4% for the B3 and S2 genomes, respectively. RASTtk found 6,991 (B3) and 6,860 (S2) protein-coding genes in the genomes. However, only 36.2% (B3) and 34.1% (S2) of these genes are functionally annotated in the RASTtk database and were successfully mapped to 370 (B3) and 362 (S2) subsystems (Table 1). There were ∼46 tRNA, ∼8 rRNA, and ∼233 pseudogenes in both genomes, as well as four ncRNAs. In B3, 62 genes were predicted to be involved in nitrogen metabolism, 32 in nitrogen fixation, 1 in nitrosative stress, 11 in ammonia assimilation, and 18 in the denitrification. In S2, 67 genes are predicted to be involved in nitrogen metabolism; 2 are engaged in cyanate hydrolysis, 32 in nitrogen fixation, 1 in nitrosative stress, 11 in nitrogen assimilation, and 20 in denitrification process (Table 1). Symbiotic genes are distributed on the symbiotic plasmids and symbiotic islands on both B3 and S2 genomes. Three genes were located on the chromosome in the nitrogen fixation category, 24 on the symbiotic plasmid 3, and five on the two non-symbiotic plasmids 1 and 4. There were two clusters on the symbiotic plasmid 3, i.e., an 18-kb segment at position 308-18401 and a 97-kb segment at position 348729-445938 (Table 2).

**TABLE 1:** Subsystem category enrichment analyses of the B3 and S2 genomes.

Categories	B3	S2
Cofactors, vitamins, prosthetic groups, and pigments	185	193
Cell wall and capsule	43	40
Virulence, disease, and defense	69	70
Potassium metabolism	13	13
Membrane transport	128	114
Iron acquisition and metabolism	18	18
RNA metabolism	49	49
Nucleosides and nucleotides	99	99
Protein metabolism	185	196
Motility and chemotaxis	76	74
Regulation and cell signaling	74	79
Secondary metabolism	5	5
DNA metabolism	143	139
Fatty acids, lipids, and isoprenoids	83	88
Nitrogen metabolism	62	67
Dormancy and sporulation	1	1
Respiration	165	166
Stress response	109	108
Metabolism of aromatic compounds	44	52
Amino acids and derivatives	412	419
Sulfur metabolism	7	7
Phosphorus metabolism	29	28
Carbohydrates	372	350
Miscellaneous	30	28

**TABLE 2:** Distribution of symbiotic genes in B3 and S2 genomes.

Chromosome	4453223-4453972	*fix*A, *fix*B, and *nif*U
Plasmid 1	185624-274785	*fix*H_1, *fix*H_2, and *nfe*D
Plasmid 3	308-18401 (cluster 1)	*nif*K, *nif*D, *nod*A, *nod*D2, and *nod*D3
	348729-445938 (cluster 2)	*nif*A, *nif*B, *nif*E, *nif*H, *nif*N, *nif*Q, *nif*S, *nif*T, *nif*W, *nif*X, *nif*Z, *nod*B, *nod*C, *nod*D1, *nod*I, *nod*Z, nolE, *fix*B, and *fix*A
Plasmid 4	637205-638909	*fix*A and *fix*B

To establish a symbiotic relationship between rhizobia and legumes, rhizobia secrete effector proteins into the host cells’ cytoplasm through various secretion systems. In the B3 and S2 genomes, type 2 (T2SS) and type 4 (T4SS) secretion systems were detected. Twenty genes of the T2SS (two *pilin*, *tad*V, two *rcp*C, two *rcp*A, *hyp*1, t*ad*Z, two *hyp*4, two *tad*A, two *tad*B, two *tad*C, *tad*D, *cpa*D, and *hyp*5) were detected in the widespread colonization island encompassing the *Tad*-locus on the chromosome, plasmid 1, and plasmid 4 in both B3 and S2. T4SS consisted of *vir* genes (*vir*B1-11, *vir*C1-C2, *vir*D1-4, *vir*AG, and *vir*E2), 13 *pvir* plasmid genes, and 11 for conjugative transfer proteins (*trb*B-L). T4SS genes are found on symbiotic plasmid 3 and plasmid 4.

### Comparative Genomics

#### Pangenome Analysis

For the pangenome analyses, the genomic data from B3 and S2 were compared with *R. phaseoli*, *R. etli*, *R. leguminosarum* bv. trifolii, *R. grahamii*, *R. tropici*, *R. esperanzea*, *R. laguerreae*, *R. leguminosarum* bv. viciae, *R. sullae*, *R. favelukesii*, *R. pusense*, *R. meliloti*, and *A. tumefaciens* as an outgroup. Pangenome analyses of the two isolates in comparison to these reference strains identified 12,768 clusters of orthologous groups. The core genome (genes present in all taxa) had 1,867 (11.6%) gene clusters. The soft-core genome (genes present in at least 95% of all taxa in consideration) had 2,104 (13.1%) gene clusters. The shell genome (moderately conserved genes present in several taxa) had 3,481 (21.6%) gene clusters, and the cloud genome (rare genes present only in a few taxa) had 8,632 (53.7%) gene clusters. The observation that only 13.1% of genes were conserved in at least 95% of the taxa while a majority (53.7%) was either identified in only one or two genomes indicates that rhizobium genera members are highly diversified.

##### Orthologous Analysis of Symbiotic Genes

Nitrogen fixation genes (*nif*) consist of 14 orthologous groups (*nif*A, *nif*B, *nif*D, *nif*E, *nif*H, *nif*K, *nif*N, *nif*Q, *nif*S, *nif*T, *nif*U, *nif*W, *nif*X, and *nif*Z). Nodulation (*nod*) genes consisted of 11 gene clusters (*nod*A, *nod*B, *nod*C, *nod*D, *nod*F, *nod*I, *nod*L, *nod*N, *nod*Q, *nod*U, and *nod*Z), and fixation (*fix*) genes of 11 gene clusters: three *fix*A; two genes for *fix*B, *fix*H, and *fix*J; and one cluster for *fix*L and *fix*Q. We observed a 100% conservation of both *nif* and *fix* genes in the genomes of isolates B3 and S3. However, of the 11 *nod* gene clusters detected in the 15 strains, only 81.8% were conserved in isolates B3 and S2 (Supplementary Table 1). Like in other common bean-nodulating rhizobia, the *nod*F and *nod*Q genes were missing in B3 and S2, but they were present in *S. meliloti*, probably for conferring host specificity to this rhizobia.

The *nod*D genes *nod*D1, *nod*D2, and *nod*D3 code for LysR-family transcriptional regulators and are highly homologous to each other. These genes in B3 and S2 were phylogenetically similar to those of other common bean-nodulating rhizobia but only distantly related to non-common bean-nodulating rhizobia. These differences among rhizobia might contribute to nodulation specificity with hosts from different legume families. The *nodU* and *nodZ* clusters in B3 and S2 might also contribute to host specificity. They were only found in strains that nodulate *P. vulgaris* (e.g., *R. etli*, *R. tropicii*, *R. phaseoli*, *R. grahamii*, and *R. esperanzeae*). The *nod*L gene, which was only detected in *S. meliloti*, is highly homologous to the non-symbiotic maltose-O-acetyl transferase genes in B3 and S2. This might indicate that the O-acetyl transferase gene evolved from *nod*L by duplication in *S. meliloti*. Another example for a gene duplication event in *S. meliloti* provides the evolution of *nod*N. Although *nod*N was absent in B3 and S2, a homolog of *nod*N, coding for a non-symbiotic MaoC dehydratase, was present in the two strains.

There were four homologous genes in the isolate S2’s *nif*H cluster; three were nitrogenase reductase genes, and one was an AAA family ATPase gene. AAA family ATPases are non-symbiotic genes that constitute a large protein family with multiple cell functions, including cell cycle regulation, organelle biogenesis, protein proteolysis disaggregation, and intracellular transport. Still, none of them have a direct role in rhizobia symbiosis. The close relationship of the AAA family ATPase gene to other *nif*H genes might also indicate that they arose from gene duplication. However, only one *nif*H homolog was found in the B3 genome.

Interestingly, the *fix* gene clusters showed a very different evolutionary trend compared to the evolution of *nod* and *nif* genes. We consistently observed various functionally similar genes forming independent gene clusters. For example, there were three clusters for *fix*A and *fix*B genes in both isolates B3 and S2. The *fix*A genes were phylogenetically different and in different genomic locations on the chromosome and the plasmids 1 and 4 of both strains (Table 2). This might point to convergent evolution (speciation events) of the *fix* genes in both strains.

##### Phylogenomic Relationship of the Isolates

**FIGURE 5:**
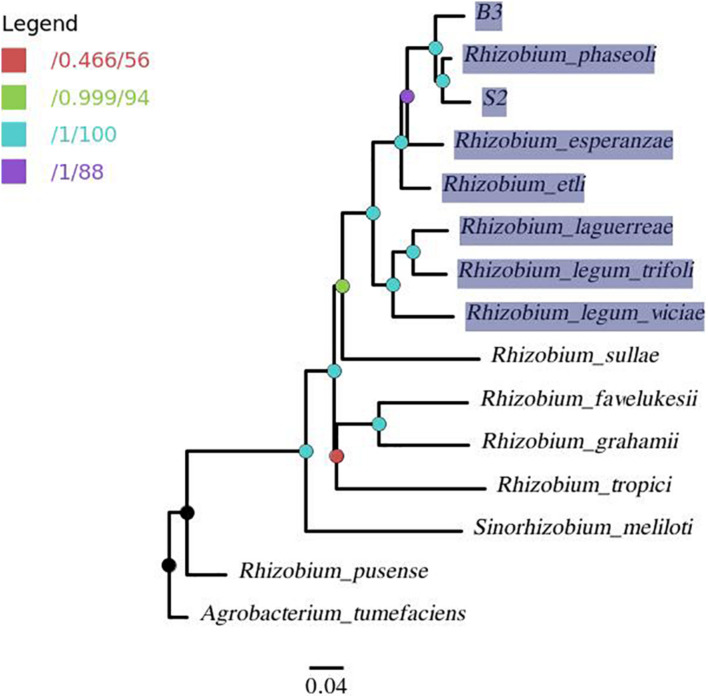
A pangenome tree showing phylogenomic relation between isolates B3, S2, and 12 reference genomes with *A. tumefaciens* as outgroup. The nodes are colored per the legend in which the first value corresponds to approximate Bayes branch support values, and the second is the UFBoot support values. The node with less than 95% support (red node) was collapsed. The highlighted taxa represents common bean-nodulating rhizobia.

A phylogenomic tree (Figure 5) from the pangenome matrix indicated that B3 and S2 share a close phylogenomic relationship with other common bean-nodulating rhizobia (highlighted taxa). *R. phaseoli* seems to be the closest to isolate S2 as they form a common sub-clade. Further, the two have the last common ancestor with isolate B3 and share a close phylogenetic relationship with *R. esperanzae*.

##### Orthologous Relationship Between Isolates B3, S2, and Other Three Closely Related Rhizobia

**FIGURE 6:**
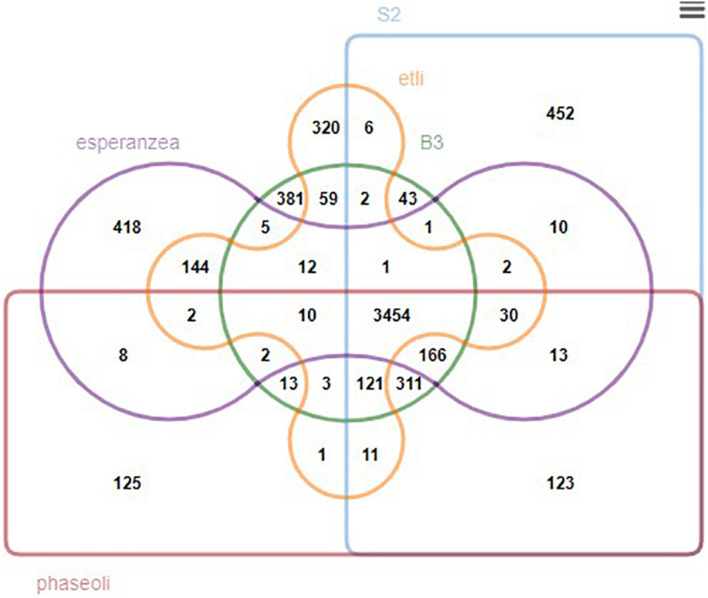
A Venn diagram showing the overlapping orthologous genes between isolates B3, S2, and three other reference rhizobia species.

We further generated a Venn diagram to show the orthologous relationship between isolates B3, S2, and three closely related rhizobia, *R. phaseoli*, *R. etli*, and *R. esperanzae* (Figure 6). We found 3,662 conserved genes between B3 and *R. etli*, 4,080 with *R. phaseoli*, and 3,651 with *R. esperanzae*. Three thousand six hundred and twenty-seven genes were conserved between S2 and *R. etli*, 4,229 with *R. phaseoli*, and 3,677 with *R. esperanzae*. The new isolates share more conserved genes with *R. phaseoli* than any other species analyzed in this study. We postulated that the two isolates and *R. phaseoli* belong to the same species. To test this hypothesis, we further performed a species delimitation analysis.

#### Species Delimitation

We computed ANI, AAI, and dDDH for the new isolates and *R. phaseoli* (Table 3). ANI and AAI values greater than 95% [with orthologous fraction (OF) ≥ 0.7] mean that they belong to the same species. Digital DNA–DNA hybridization, based on genome blast distance phylogeny, mimics laboratory-based DNA–DNA hybridization, and dDDH values ≥70% indicates that the two organisms belong to the same species. These tests confirmed that isolates B3 and S2 are different strains of *R. phaseoli* (ANI ≥ 95%, AAI ≥ 95%, and dDDH ≥ 70%).

**TABLE 3:** ANI, AAI, dDDH, and genetic distances between isolates B3, S2, and *R. phaseoli*.

Query genome	Reference genome	AAI	ANI	dDDH	Distance
B3	*R. phaseoli*	99.18	98.97	91.8	0.0102
S2	*R. phaseoli*	99.8	99.78	98.9	0.0019

*Orthologous fraction (OF) was put at 0.8 for ANI and AAI. Abbreviations: AAI, average amino acid identity; ANI, average nucleotide identity; dDDH, digital DNA–DNA hybridization.*

#### Synteny Analysis

**FIGURE 7:**
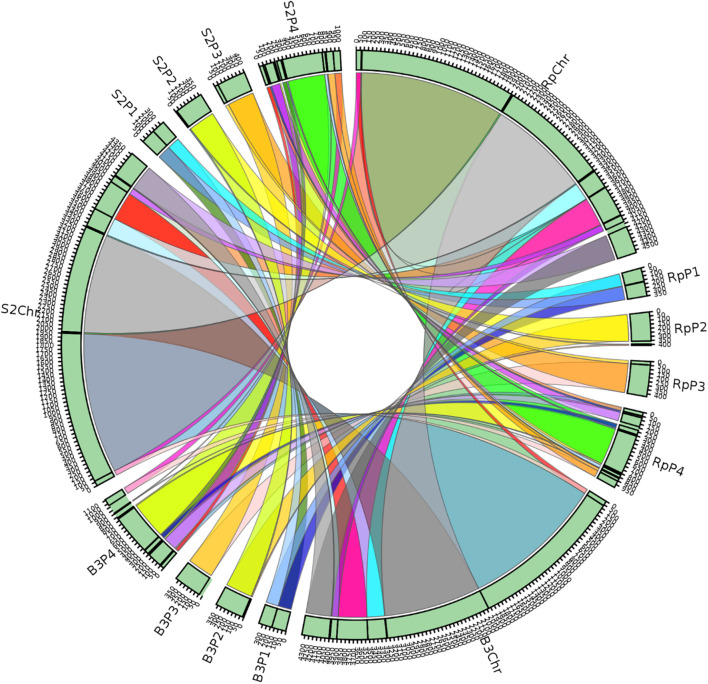
Synteny blocks between the isolates B3, S2, and *R. phaseoli*. Rp, *R. phaseoli*; Chr, chromosome; P1–P4, plasmids 1–4.

A synteny block is a region in a genome spanning a series of orthologous genes co-arranged with another genome sequence with either the same or different orientation or loci. A synteny analysis provides a framework in which duplication and orientation of homologous genes are studied. Sibelia, a tool based on iterative de Bruijn graphs, can find not only synteny blocks between closely related microbial genomes but also repeated blocks within the same genome. We found 11 synteny blocks on the chromosomes of *R. phaseoli*, B3, and S2; two synteny blocks on each of the plasmids 1–3; and 12 synteny blocks on plasmid 4 (Figure 7). The majority (51.7%) of the synteny blocks show vital conservation of genomic sequences in *R. phaseoli* and isolates B3 and S2. These blocks were found in the same loci and the same orientation. However, 48.3% of them have either translocated to different loci, inverted, or were missing (Table 4).

**TABLE 4:** An analysis of synteny blocks between *R. phaseoli* and isolates B3 and S2.

Genome location	Block	Locus	Synteny block rearrangement	Evolutionary event
Chromosome (11 blocks)	1	70091-63806	Inverted duplication at 3638832-3644956 and 4032266-4038731 in *R. phaseoli*	Inversion and duplication in *R. phaseoli*
			Absent in B3 and S2	Deletion in B3 and S2
	2	2009891-2027795	Absent in B3	Deletion in B3
	3	3389682-3404271	Absent in B3	Deletion in B3
	4	3989983-4000450	Absent in *R. phaseoli*	Deletion in *R. phaseoli*
Plasmid 1 (2 blocks)	1	172356-352129	Translocation 1-179755 in B3 and S2	Translocation in B3 and S2
	2	1-166278	Translocation to 179756-346033 in B3 and S2	Translocation in B3 and S2
Plasmid 2 (2 blocks)	1	1-357870	Translocation to 16820-375979 in B3 and S2	Translocation in B3 and S2
	2	396652-413457	Translocation to 1-16806 in B3 and S2	Translocation in B3 and S2
Plasmid 3 (2 blocks)	1	681-44285	Absent in B3	Deletion in B3
Plasmid 4 (12 blocks)	1	796101-811343	Absent in S2	Deletion in S2
	2	179531-197755	Translocated to 239924-258137 in B3	Translocation in B3
			Absent in *R. phaseoli*	Deletion in *R. phaseoli*
	3	45545-67679	Absent in B3	Deletion in B3
	4	285455-301543	Absent in B3	Deletion in B3
	5	820724-857199	Absent in B3	Deletion in B3

*Only blocks with possible evolutionary changes are shown.*

## Discussion

Common bean is undoubtedly one of the primary protein sources in developing countries (Castro-Guerrero et al., 2016). Its cultivation can immensely benefit from rhizobia inoculation by limiting the application of disastrous and expensive chemical fertilizers. However, for large-scale agricultural applications, inoculants well-adapted to the local soil conditions are necessary. Like soils in many other countries along the equator, Western Kenyan soils are characterized by pH values as low as 4.6 and often deficient phosphate concentrations. Besides, common bean suffers from high aluminum concentrations, which can be up to 9.4 Cmol/kg (Wekesa et al., 2016). Foreign rhizobia often fail to survive or do not effectively nodulate with their hosts or perform insufficient nitrogen fixation under these harsh conditions. Formulations with successful inoculants require a clear understanding of strain efficacy, adaptability to the target environment, and knowledge about the bacteria’s physical–chemical and genetic characteristics (Stephens and Rask, 2000; Xavier et al., 2004). To provide rhizobia suitable for consideration as inoculants for Western Kenyan soils, we searched for indigenous isolates and compared their symbiotic features with those of the commercially available CIAT899 inoculum used in Kenyan agriculture. For two promising candidates, physicochemical properties and genome data are provided.

### Symbiotic Efficiency

Initial studies with the two indigenous isolates, B3 and S2, clearly showed that they are rhizobacteria with nitrogen fixation ability (Figure 1). Inoculated common bean plants possess greener leaves and had more biomass, nitrogen, chlorophyll, and carotenoid content than plants inoculated with CIAT899 on nitrogen-limiting conditions. Also, Ouma et al. (2016) showed that native indigenous rhizobia displayed higher symbiotic ability than commercial strains, such as CIAT899, because they are better adapted to Kenya’s local conditions. The inoculated plants’ overall phenotype compared to nitrogen-fertilized plants may suggest that—apart from fixing nitrogen—there might be other benefits for the host, such as secretion of phytohormones and relieving the plants from abiotic stress (Kevin Vessey, 2004; Hungria et al., 2013). Both strains produce higher amounts of IAA than the commercial CIAT899 inoculant (Figure 2). Rhizobacteria-produced IAA improved plant growth by several mechanisms and became an important selection criterion for the choice of commercially used inoculum (Alemneh et al., 2020; Keswani et al., 2020). A striking observation was that the isolate B3 was more efficient in phosphate solubilization in the culture media than CIAT899. This is an essential feature for rhizobia application, although its contribution to plant performance is less investigated (Concha and Doerner, 2020).

### Phenotypic and Biochemical Characteristics

Commercial isolates such as CIAT899 suffer under acid soil conditions in Western Kenya, and CIAT899 was highly inhibited in the media with pH 4.8. In contrast, the indigenous isolates B3 and S2 grew in these conditions. Furthermore, B3 and S2 released acidic substances into the media, as shown by the color change of the pH indicator. Although early researchers (Jensen and Hansen, 1968) believed that acid-producing rhizobia are sensitive to acidity, later studies found this was not always the case. For instance, Cooper (1982) found that six of his acid-producing lotus rhizobia were more tolerant to acidic conditions than alkaline-producing rhizobia. This demonstrates that alkaline substance release is not a prerequisite for bacterial survival under acidic environmental conditions. We suggest that the isolates B3 and S2 have adapted their metabolic activities to the acidic environment in Western Kenyan soils. Although the biochemical and molecular basis is unknown, acid-producing bacteria grow fastest under those abiotic stress conditions (Boakye et al., 2016). Thus, they may have advantages for agricultural applications over the commercial inocula used in Kenya today and worldwide in countries with acidic soils.

Dark-grown bacteria’s inability to absorb Congo red has been used as a rhizobia-identification strategy (Nyaga and Njeru, 2020). In earlier reports, *R. leguminosarum*, *R. trifoli*, and *S. meliloti* absorbed Congo red (Kneen and LaRue, 1983), while the B3 and S2 isolates only partially absorbed the dye, similar to reports from Kawaka et al. (2014) for other isolates. Therefore, the use of Congo red for rhizobia indication may not be reliable, especially in the early stages of their cultivation process. However, other morphological characters specific for rhizobia were observed for B3 and S2, such as the milky white and mucoid structure, the rod-shaped and convex elevation of the colonies, and their classification as gram-negative bacteria upon staining, as reported by Somasegaran and Hoben (1994).

### Protein Secretion System

Bacterial survival depends on their interactions with the environment; hence, they have evolved various mechanisms to secrete proteins (Green and Mecsas, 2016). Many secretion systems generally exist in prokaryotes, but only seven have been studied in detail (Tseng et al., 2009). Isolates B3 and S2 have T2SS and T4SS. T2SS is thought to transport various proteins and other compounds out of the cell (Green and Mecsas, 2016). Additionally, the *Tad* genes in the widespread colonization island were essential in forming adhesive pili in the genera *Actinobacillus* (Tomich et al., 2007). Therefore, it is probable that T2SS in rhizobia is involved in the formation of adhesive substances necessary for its attachment to the root hairs of its host.

T4SS, which is homologous to T3SS and T6SS, has been shown to transport effector proteins into the host cell’s cytoplasm. These effector proteins suppress the host immune system (Masson-Boivin et al., 2009) by disrupting nodulation autoregulatory processes and promoting nodulation in the host roots (Nelson and Sadowsky, 2015). The *vir*B1-11 and *vir*D4 genes of T4SS were identified in both B3 and S2. T4SS appear to operate also in *R. phaseoli*, *R. etli*, *R. leguminosarum*, *R. pusense*, *S. meliloti*, *Mesorhizobium loti*, and *Sinorhizobium medicae* (cf. Hubber et al., 2007; Sugawara et al., 2013); however, essential genes for T4SS are absent in *R. grahamii* and *R. tropici* CIAT899.

Interestingly, the genes for this secretion system are located on the symbiotic plasmids. Therefore, the T4SS secretion system’s exact function in the symbiotic interaction of B3 and S2 with common bean needs to be investigated in the future. It might be interesting to see how T4SS influences symbiosis under acidic soil conditions in Kenya.

### Symbiotic Genes

An orthologous analysis suggests that gene duplication seems to be the mechanism that led to the evolution of *nod* genes in rhizobia. The *nodD* gene homologs recovered from the genomes of B3 and S2 were highly similar to other *nodD* genes from common bean-nodulating rhizobia and distantly related to others like the *Medicago* sp*.-*nodulating *S. meliloti*. However, as members of the LysR transcriptional regulator family (Peck et al., 2006), all these genes are phylogenetically similar to other members of this family that have no role in the legume–rhizobia symbiotic establishment. This might indicate the possibility of a duplication event among members of LysR regulators that later adapted differently to recognize specific flavonoids from legumes to regulate rhizobia–host specificity during legume–rhizobia symbiosis (Göttfert, 1993; Smith et al., 2015; Liu et al., 2018).

The nodL protein, a member of O-acetyl transferases, is implicated in the acetylation of nod factors (Bloemberg et al., 1994) in *R. leguminosarum* and *S. meliloti*. The nodL protein sequence is highly similar to sequences of other acetyltransferases with no function in symbiosis. In particular, nodL from *S. meliloti* is phylogenetically similar to the maltose-O-acetyl transferase from isolates B3 and S2. This protein acetylates glucose and maltose exclusively at the C6 position of the non-reducing end at the glucosyl moiety (Lo Leggio et al., 2003). Downie (1989) showed that the *R. leguminosarum nod*L gene was homologous to the acetyltransferase genes *lac*A and *cyc*E.

Another example is the *nodN* gene in *S. meliloti*, homologous to MaoC family dehydratase genes detected in all other isolates investigated in this study, including B3 and S2. Most genes for MaoC domain-containing proteins are part of an operon involved in synthesizing monoamine oxidase, and proteins are involved in producing the root hair deformation (HAD) factor, specifically on *Medicago*. Therefore, it is possible that nodN belongs to the MaoC dehydratase family and has arisen by duplication in *Medicago*-nodulating rhizobia.

In contrast to *nodD*, the *fix* genes seem to have arisen from independent evolution events (speciation). Although there were multiple *fix*A, *fix*B, and *fix*H genes in the two isolates’ genomes, they were phylogenetically unrelated. This existence of functionally related non-homologous genes in the same genome may point to convergent evolution from unrelated genes instead of duplication events. One of the *fix*A clusters with no homology to any other rhizobial gene cluster was identified in B3 and S2. This may point to the possibility of a region-specific independent evolution of *fix* genes.

### Phylogenomics and Species Delimitation

Phylogenomics and species delimitation analysis classified the two isolates B3 and S2 as members of *R. phaseoli*. However, the three genomes’ syntactic analysis showed possible differences in the gene order, conservation, and orientation between *R. phaseoli* and B3 and S2. For example, seven synteny blocks in *R. phaseoli* were absent in isolate B3 and two in S2. This might indicate that these regions were either lost in the genomes of B3 and S2 or inserted in the genome of *R. phaseoli*. We also noticed that five and four synteny blocks in B3 and S2, respectively, were translocated to different sites, as seen in *R. phaseoli*. Considering that these regions are long DNA sections with many genes, this can significantly impact microbial phenotype variation (Darmon and Leach, 2014). Previously, the translocation of genes was shown to modulate expression levels of prokaryotic constitutive genes due to changes in chromosomal positions (Block et al., 2012). These changes in the indigenous isolates might allow better adaption to the local edaphic factors. Genome deletion has been described in the past as one of the mechanisms of bacterial adaptation to harsh environments (Koskiniemi et al., 2012).

In conclusion, we identified two indigenous rhizobial strains in Western Kenya’s acidic soils that nodulate with the common bean. Compared to inoculation with the commercial rhizobial strain CIAT899, the indigenous rhizobia perform better under controlled greenhouse conditions. Physiological characterization of the rhizobial strains uncovered that they slightly differ; however, growth in acidic media suggests that they might be better adapted to Kenya’s soil conditions. Genome analyses identified them as different strains of *R. phaseoli*.

## Data Availability Statement

The datasets presented in this study can be found in online repositories. The names of the repository/repositories and accession number(s) can be found below: https://www.ncbi.nlm.nih.gov/Traces/wgs/JAAVVP01?display=contigs and https://www.ncbi.nlm.nih.gov/Traces/wgs/JAAVVN01?display=contigs.

## Author Contributions

CW performed the statistical analysis and wrote the first draft of the manuscript. RO and AF wrote sections of the manuscript. All authors contributed to the conception and design of the study, manuscript revision, read, and approved the submitted version.

## Conflict of Interest

The authors declare that the research was conducted in the absence of any commercial or financial relationships that could be construed as a potential conflict of interest.

## Publisher’s Note

All claims expressed in this article are solely those of the authors and do not necessarily represent those of their affiliated organizations, or those of the publisher, the editors and the reviewers. Any product that may be evaluated in this article, or claim that may be made by its manufacturer, is not guaranteed or endorsed by the publisher.
