# Supplementary material for: Isolation and Characterization of High-Efficiency Rhizobia From Western Kenya Nodulating With Common Bean
Source: Front Microbiol. 2021 Sep 10;12:697567. doi: 10.3389/fmicb.2021.697567 (PMC8461304; doi:10.3389/fmicb.2021.697567)
Supplement: Supplementary file 1 [file Data_Sheet_1.docx]

***Supplementary Material***

**Supplementary Table 1**. Conservation of *nif, nod* and *fix* genes in respective rhizobia strains.

| Rhizobia strains | Percent *nif* genes | Percent *nod* genes | Percent *fix* genes |
| --- | --- | --- | --- |
| B3 | 100 | 81.81818 | 100 |
| S2 | 100 | 81.81818 | 100 |
| *Rizobium phaseoli* | 100 | 81.81818 | 100 |
| *Rizobium etli* | 100 | 81.81818 | 100 |
| *Rizobium tropici* | 100 | 81.81818 | 100 |
| *Rizobium leguminosarum bv trifoli* | 71.42857 | 72.72727 | 81.81818 |
| *Rizobium leguminosarum bv viciae* | 64.28571 | 72.72727 | 100 |
| *Sinorhizobium meliloti* | 71.42857 | 81.81818 | 100 |
| *Rhizobium grahamii* | 100 | 90.90909 | 100 |
| *Rhizobium pusense* | 7.142857 | 54.54545 | 100 |
| *Rhizobium esperanseae* | 71.42857 | 63.63636 | 81.81818 |
| *Rhizobium laguarrae* | 64.28571 | 72.72727 | 81.81818 |
| *Rhizobium sullae* | 28.57143 | 36.36364 | 81.81818 |
| *Rhizobium favelukesi* | 100 | 81.81818 | 81.81818 |

**
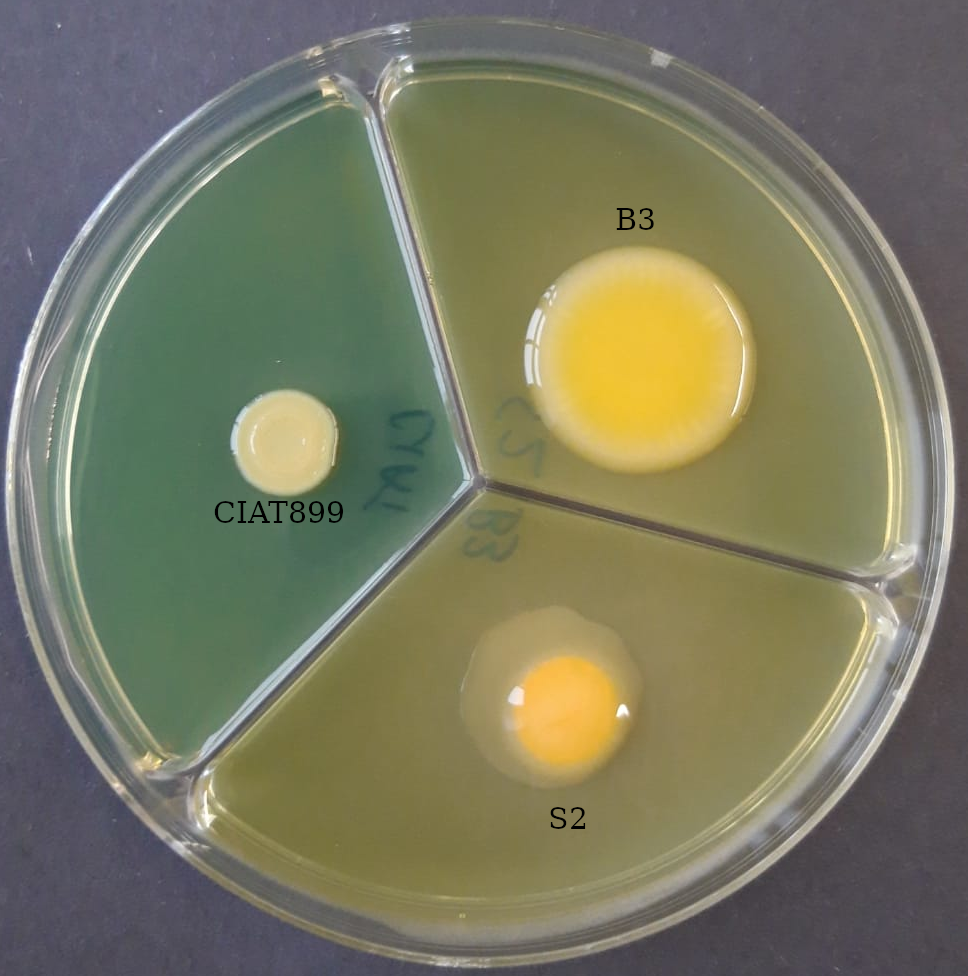
Supplementary Figure 1**. Bromothymol blue (BTB) reaction test. Acid producing bacteria (B3 and S2) turns blue BTB to yellow while alkaline producing bacteria (CIAT899) turns it to green.

**
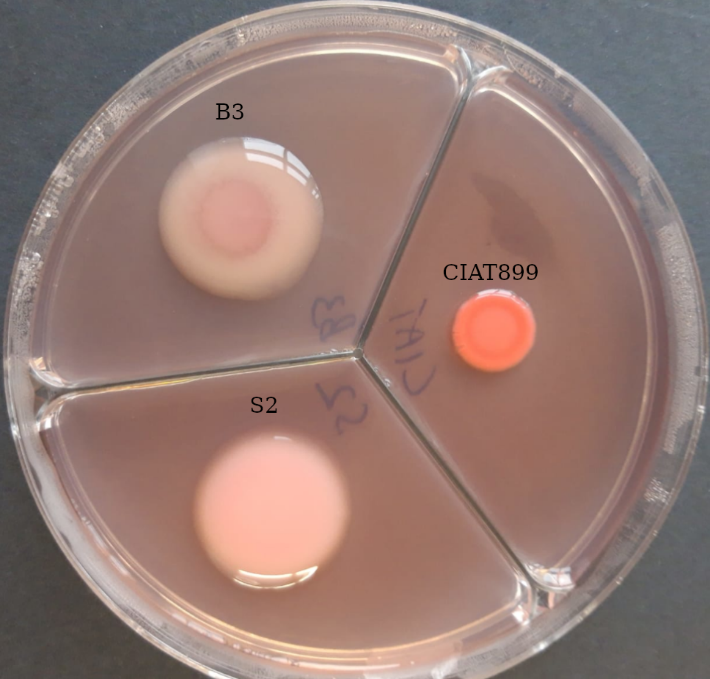
**

**Supplementary Figure 2**. Congo red absorption test. B3 and S2 only slightly absorbs congo red.

**
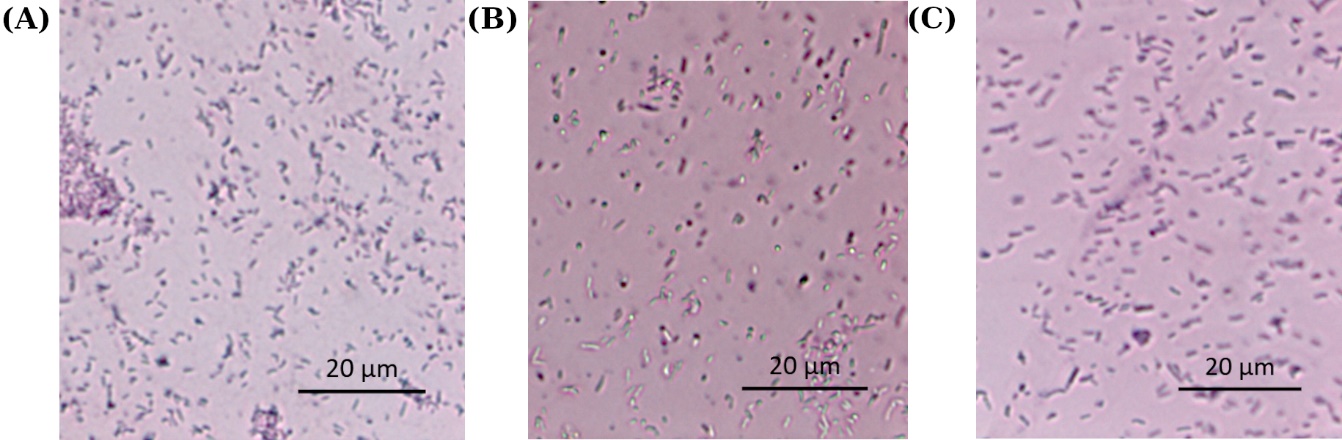
**

**Supplementary Figure 3**. Gram staining test. Gram-negative bacteria (all isolates) turns red on staining.
